# Parasitic contamination and public health risk of commonly consumed vegetables in Ibadan-Nigeria

**DOI:** 10.11604/pamj.2020.36.126.19364

**Published:** 2020-06-25

**Authors:** Oluwasola Olaiya Obebe, Olufemi Oludare Aluko, Olufarati Oludunsin Falohun, Kayode Blessing Akinlabi, ThankGod Emmanuel Onyiche

**Affiliations:** 1 Department of Veterinary Parasitology and Entomology, University of Ibadan, Ibadan, Nigeria,; 2 Department of Community Health, College of Health Sciences, Obafemi Awolowo University, Ile Ife, Nigeria,; 3 Department of Veterinary Physiology, Biochemistry and Pharmacology University of Ibadan, Ibadan, Nigeria,; 4 Department of Veterinary Parasitology and Entomology, University of Maiduguri, Maiduguri, Nigeria

**Keywords:** Parasites, vegetables, contamination, Ibadan-Nigeria

## Abstract

**Introduction:**

vegetables form a major component of the human diet. However, poor agronomic practices may put consumers at risk of parasitic infections. This study evaluated the parasitic contamination of vegetables grown in selected farms in Ibadan, Nigeria.

**Methods:**

Two hundred and eighty vegetable species: African eggplant (*Solanum macrocarpon*), lettuce (*Lactuca sativa*), cucumber (*Brassica oleracea*), spinach (*Amaranthus cruentus*), white jute (*Corchorus olitorius*), pumpkin (*Telfaria occidentalis*), green pepper (*Capsicum sp*.), okro (*Abelmoschus esculentus*), quill grass (*Celosia argenta L*), tomato (*Lycopersicum sativus*) were collected from farms within Ibadan. Samples were washed in water, and the resulting washing solution was filtered and centrifuged to concentrate the parasitic stages. Sediments were examined by iodine and modified Ziehl-Neelsen stained smears technique.

**Results:**

parasites were detected in 14 (5.0%, 95% CI 32.6%-67.3%) of samples. The highest contaminated vegetable was white jute 32.1 (95% CI 17.9%-50.6%), followed by pumpkin 7.1(95% CI 1.9-22.6), quill grass 7.1% (95% CI 1.9-22.6) and lettuce 3.5 (95% CI 0.6-17.7). The commonest parasites were *Strongyloides stercoralis* larvae 42.9 (95% CI 21.3-67.4), *Entamoeba histolytica/E.dipaar* 21.4 (95% CI 7.5-47.5), *Trichostrongylus spp* 21.4 (95% CI 21.3-67.4), and *Ascaris sp*. 14.3 (95% CI 4.0-39.9).

**Conclusion:**

these findings provide evidence of contamination of vegetables from farms in Ibadan with parasites of public health importance. Information on best practices should be designed, packaged and disseminated through appropriate channels to enhance positive behavior change among farmers.

## Introduction

The consumption of vegetables has increased in recent years because of their nutritional importance and health benefits [1]. Vegetables form a key component of a healthy diet, highly beneficial for the maintenance of health and prevention of diseases [2, 3]. However, consumption of contaminated vegetables plays a significant role in the transmission of parasitic foodborne illnesses [4, 5]. In recent times, vegetables had been shown to be contaminated with different types of enteric parasites, among which *Entamoeba histolytica, Giardia duodenalis, Cryptosporidium sp. Hymenolepis sp. Taenia sp. Ascaris lumbricoides, hookworms, Enterobius vermicularis, Trichuris trichiura, Toxocara sp*. and the genus *Trichostrongylus* have been regarded as most common [5-7]. Human Infection by these parasites can cause various clinical symptoms [8], thereby making the control of those parasites in vegetables a public health intervention priority.

Oocysts/cysts, eggs or larvae of enteric parasites can contaminate vegetables from polluted naturally composted manure, manure from grazing animals, raw, or partially treated sewage sludge, irrigation water, and wastewater from livestock operations [9, 10] and humans get infected through consumption of improperly washed or uncooked vegetables containing infective stage of these parasites [8, 11]. There may currently be an increase in parasitic diseases in human population in developing countries due to an increase in the consumption of meals in canteens, restaurants and fast food service premises, increase in the at-risk population such as the elderly, immunocompromised and children as well as changes in agronomic and vegetables processing practices [12, 13]. Several studies have reported a high prevalence of intestinal parasites on vegetables worldwide, such as found in the Philippines [14], Iran [15], Ghana [12], Kenya [16] and in Nigeria [17, 18]. However, Information on the level of contamination by parasites on vegetables from farms are lacking, especially in developing countries including Nigeria where parasitic diseases are endemic in the population. This study is therefore aimed at assessing the parasitic contamination of commonly consumed vegetables from selected farms in Ibadan metropolis.

## Methods

**Figure 1:**
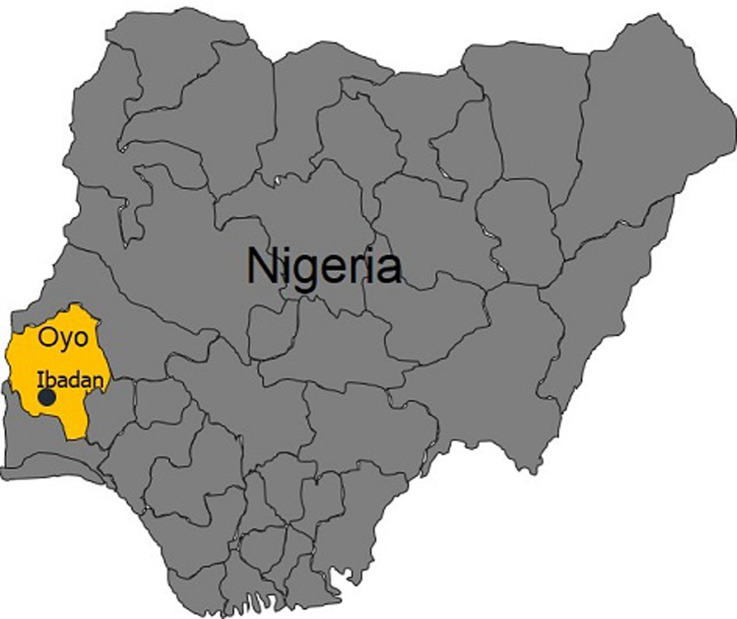
map of Nigeria showing the study area

**Study area:** this study was carried out in Ibadan city, the largest indigenous city in sub-Saharan Africa. Ibadan, the capital of Oyo State is located between longitude 70 20´ and 70 40´ East of the Greenwich meridian and between latitude 30 55´ and 40 10´ North of the equator. Climatically Ibadan falls under the tropical wet and dry climates (Koppen climate classification, Aw), with a lengthy wet season, which runs from March to October, and relatively constant temperatures throughout the year, between 23 °C and 33 °C during the dry season. Ibadan has 11 local government areas (LGAs). Five LGAs were chosen for the study (Figure 1) while fourteen commercial farms were selected from the Five LGAs. These farms were considered important as the majority of fresh vegetables sold in different markets of Ibadan metropolis were brought from them.

**Sample collection:** a total of 280 vegetables samples comprising of ten leafy vegetable species: African eggplant (*Solanum macrocarpon*), lettuce (*Lactuca sativa*), cucumber (*Brassica oleracea*), spinach (*Amaranthus cruentus*), white jute (*Corchorus olitorius*), pumpkin (*Telfaria occidentalis*), green pepper (*Capsicum sp*.), okro (*Abelmoschus esculentus*), quill grass (*Celosia argenta L*) and tomato (*Lycopersicum sativus*), were included in the study. Socio-demographic characteristic and agronomic practice variables of 30 farmers were also obtained using semi- structured questionnaire.

**Parasitological analysis of vegetables:** 200-250 g samples of each vegetable were washed in distilled water in a plastic container for the removal of parasitic ova, larva or cysts. The suspension was strained through a sterile sieve to remove undesirable materials [19] and then centrifuged at 5000 rpm for 5 minutes [18]. The supernatant was discarded while the sediment obtained was transferred unto labeled clean slides for examination under the microscope using X10 and X40 objectives [19]. Modified Ziehl-Neelsen stained smears were also prepared for detection of coccidian oocysts [20]. A pictorial species identification guide from “District Laboratory Practice in Tropical Medicine” was used. Parasites were recorded as either present or not.

**Data analysis:** data entry and analysis were carried out using SPSS version 21.0 (SPSS Inc. Chicago, IL, USA). Frequency tables and percentages were used to display categorical data. The 95% confidence intervals were constructed around the identified levels of contamination.

## Results

**Figure 2:**
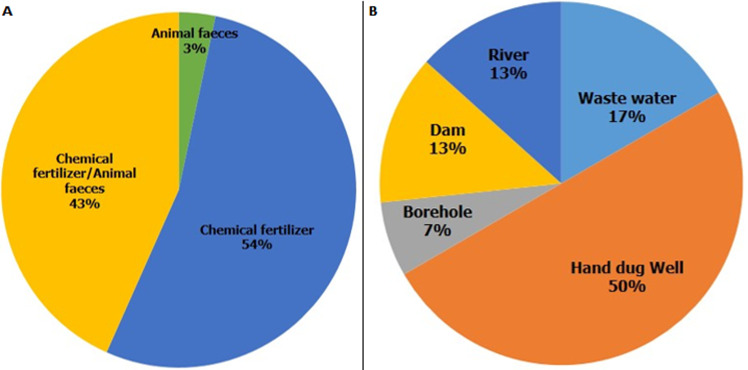
agronomic practices of vegetable farmers (A,B)

**Figure 3:**
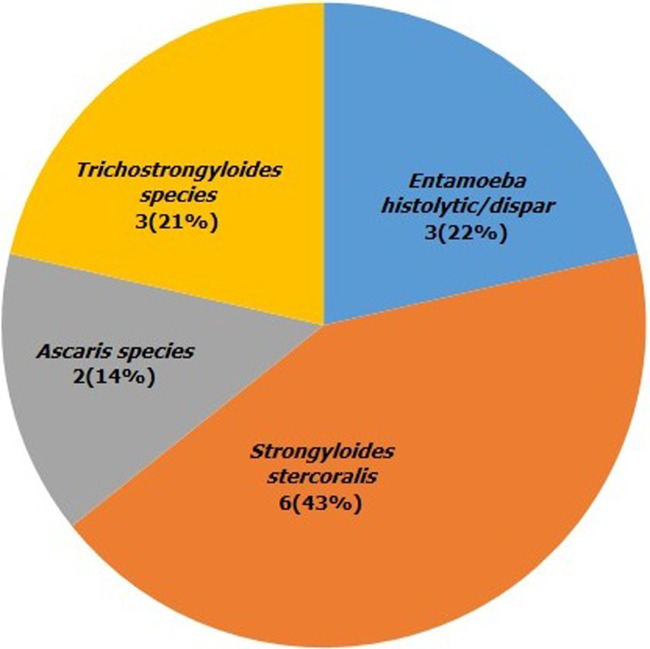
distribution of parasites identified from leafy vegetable samples in Ibadan

**Socio-demographic characteristics of respondents:** the result indicates that age composition of the farmers ranged from a minimum of 23 to a maximum of 75 years with a mean of 41.6 years and standard deviation of 12.027. There are more educated farmers than non-educated and more males (80%) than females (20%). About 90% of the respondents are married while only 10% are single. More farmers 19(63.3%) were full-time vegetable growing farmers rather than part-time 11(36.7%). (Table 1). Agronomic Practices of vegetable farmers in Ibadan showed that majority of them (54.0%) use chemical fertilizers while 3% of the farmers use animal feces as fertilizer. Only 5(17.0%) of farmers irrigate their farms with wastewater while half 15(50.0%) of them uses water from hand dug well. (Figure 2, Figure 3).

**Table 1:** socio demographic characteristics of vegetable farmers in Ibadan, Nigeria

Variables	Frequency (%)
**Age (years)**	
20-39	12 (40.0)
40	15 (50.0)
60-79	3(10.0)
**Sex**	
Male	24 (80.0)
Female	6 (20.0)
**Marital status**	
Married	27 (90.0)
Single	3 (10.0)
**Education**	
Educated	27 (90.0)
No education	3(10.0)
**Vegetable cultivation as an occupation**	
Full tim	19 (63.3)
Part time	11 (36.7)
**Years of farming experience (yrs)**	
< 10	24(80.0)
11-20	4(13.3)
>20	2(6.7)

This study showed that fourteen (5.0%, 95% CI 32.6%-67.3%) samples were contaminated with intestinal parasites with white jute (32.1%, 17.9-50.6) being the highest contaminated vegetable, followed by pumpkin (7.1%, 1.9-22.6), quill grass (7.1%, 1.9-22.6) and lettuce as the least contaminated (3.5%, 0.6-17.7). No parasites were detected in African eggplant, cucumber, pepper, okra and tomato (Table 2). The distributions of the parasites were *Strongyloides stercoralis* larvae (42.9%, 21.3-67.4), *Entamoeba histolytica* (21.4%, 7.5-47.5), *Trichostrongylus spp* 3(21.4%, 21.3-67.4), and *Ascaris spp*. (14.3%, 4.0-39.9). Multiple contaminations of cysts of *Entamoeba histolytica*, ova of *Ascaris spp*., *Trichostrongylus spp*., and *Strongyloides stercoralis* larvae were detected in white jute and quill grass.

**Table 2:** distribution of parasites in different vegetables from selected farms in Ibadan, Nigeria

Vegetables	Number ½examined	No(%) positive	95% CI	Parasites encountered
Solanum macrocarpon (African eggplant)	28	0 (0)	-	ND
				
Lactuca sativa (lettuce)	28	1 (3.5)	0.6-17.7	Strongyloides stercoralis
Brassica oleracea (cucumber)	28	0 (0)	-	ND
Amaranthus cruentus (spinach)	28	0 (0)	-	ND
				
Corchorus olitorius (white jute)	28	9 (32.1)	17.9-50.6	Entamoeba histolytica/E.dispar Ascaris sp., Trichostrongylus sp., Strongyloides stercoralis
Telfaria occidentalis (pumpkin)	28	2 (7.1)	1.9-22.6	Strongyloides stercoralis
Capsicum (pepper)	28	0 (0)	-	ND
Abelmoschus esculentus (pkra)	28	0 (0)	-	ND
Celosia argenta L (quill grass)	28	2 (7.1)	1.9-22.6	Ascaris suum, Trichostrongylus spp
Lycopersicum sativus (tomato)	28	0 (0)	-	ND
Total	280	14(5.0)	32.6-67.3	ND

Key: ND= Not detected

## Discussion

The study assessed the parasitic contamination of commonly consumed vegetables from selected commercial vegetable farms in Ibadan metropolis. In addition, Socio-demographic characteristic and agronomic practices of farmers were also obtained. The age of the farmers engaged in vegetable farming in Ibadan metropolis falls within the economically active age group when they can productively carry out the rigor of farming. The distribution of the respondents by sex shows that male involvement in farming is high compared to females. This is an indication of the patriarchal head of the family where more men than women are involved in farming especially in Yoruba land in order to provide food and other essential needs of their households. While more of the farmers engaged in vegetable farming as a full-time occupation rather than part-time, their farming experience also revealed that most of the farmers had many years of farming experience. The implication is the present economic recession in the country has pushed more people to farm.

The examination of vegetables in the current study revealed a low, overall prevalence of 5% when compared with previous studies within Nigeria and outside Nigeria by Daryani *et al*. [21] in Ardabil city, Iran; Damen *et al*. [17] in Jos, Nigeria; Uga *et al*. [22] from Hanoi and Gharavi *et al*. [23] from Tehran. However, the prevalence is comparable with 3.5% in Northeastern Nigeria [24] and 6.3% in Turkey [25]. The low and the disparity in prevalence may be attributed to the diagnostic test used, environmental factors, geographical location [26, 27], as well as the differences in shape and surface of vegetables with uneven surfaces facilitating sticking of ova, cysts, and oocysts of parasite than smooth surfaces [17, 28]. The most frequent occurring parasite in sampled vegetables was *Strongyloides stercoralis* larvae. This is similar to the reports from Koforidua, Ghana [12] and Ibadan, Nigeria [29]. The common occurrence of *Strongyloides stercoralis* may be associated with poor sanitation where soil and water can be contaminated by human feces [12]. *Strongyloides stercoralis* have been reported as agents of diarrhoea in HIV/AIDS infected persons [30].

The detection of *Ascaris spp*. and *Trichostrongylus spp* in this study agrees with the findings of Ebrahimzadeh *et al*. [31] in Iran, Uneke [32] in Nigeria and Wafa [33] in Saudi Arabia who reported similar nematodes in their different studies. Contamination of vegetable by *Ascaris sp*. and *Trichostrongylus* is possibly due to animal and human fecal matter polluting the water supply used for irrigation [34]. *Ascaris* eggs are more resistant than other intestinal parasites due to diverse adverse environmental conditions [35], while *Ascaris suum* from pigs are known to be zoonotic [36]. Human trichostrongylosis cases have been reported sporadically from different countries [37, 38]. The detection of the only protozoan, *Entamoeba histolytica/E.dispar* in this study agrees with the findings of Benti and Gemechu [39] and Ali *et al*. [40] in Ethiopia and Saudi Arabia respectively. The occurrence of this protozoan cyst could be attributed to the contamination of fresh vegetables before harvest, either by irrigation with wastewater contaminated with human feces or directly from human feces [41, 42].

In our study, cucumber, pepper, and tomatoes were found to be free of parasites. This is consistent with the findings of other investigators who also reported the absence of parasites in leafy vegetables like cucumber, pepper and tomatoes [18, 43, 44]. Smooth surface which reduces the rate of parasitic attachment may suggest the non-contamination of these vegetables [17, 28]. Other parasites of public health importance like *Giardia intestinalis* and *Cryptosporidium parvum*, which were detected by previous studies [12, 28, 45] were not found in the present study. The reasons could be associated with varying ecological and climatic conditions, differences in methods of detection, and difference in endemicity of parasites from one area to the other. Despite the valuable information provided by this study, it is not devoid of limitations. Level of contamination of irrigation water, manure and soil in which green vegetables are cultivated on these farms were not assessed. Further studies are also needed to determine the risk factors associated with vegetable contamination in the study.

## Conclusion

Our results clearly show that raw leafy vegetables grown in farms in Ibadan metropolis are quite often contaminated with parasites. These types of vegetables should be considered as potential risk to the farming communities, handlers, transporters, and consumers of these vegetables. It is therefore recommended that information on contamination preventive practices, from farm-to fork continuum should be packaged and disseminated through appropriate channels to enhance vegetables contamination preventive behavior change among farmers.

### What is known about this topic

- Vegetables had been revealed to be contaminated with enteric parasites;
- Oocysts/cysts, eggs or larvae of parasites can contaminate vegetables through poor agronomic practices.

### What this study adds

- Parasites were detected in 5.0% vegetable sample;
- Detection of parasites of public health importance;
- Highest contaminated vegetable was White jute 32.1%.
